# Direct measurement of multi-elements in high matrix samples with a flow injection ICP-MS: application to the extended *Emiliania huxleyi* Redfield ratio†
†Electronic supplementary information (ESI) available. See DOI: 10.1039/c8ja00031j


**DOI:** 10.1039/c8ja00031j

**Published:** 2018-05-29

**Authors:** Qiong Zhang, Joseph T. Snow, Phil Holdship, David Price, Paul Watson, Rosalind E. M. Rickaby

**Affiliations:** a Department of Earth Sciences , University of Oxford , OX1 3AN , UK . Email: Joan.zhang@earth.ox.ac.uk; b PerkinElmer, Inc. , Seer Green, Buckinghamshire , HP9 2FX , UK; c Elemental Scientific Instruments Ltd. , 73 Manchester Road, Warrington , WA1 4AE , UK

## Abstract

The quotas of a limited number of trace elements in the extended Redfield ratios have been determined before and thought to reflect the requirements of phytoplankton. However, these quotas are found to be quite variable under different environmental conditions, suggesting that the cellular trace metal quota is not an accurate measure of cellular trace metal requirement. Here we present a method that has been developed and optimised for direct analysis of 32 elements simultaneously in small volume of cell lysate in buffers with a high salt matrix (800 μL, up to 30% TDS). We then demonstrate the application of the method to resolve the extended Redfield ratio of cell requirement by measuring the intracellular trace element composition of six *Emiliania huxleyi* strains isolated from different locations. The method uses a quadrupole-ICP-MS with a collision/reaction cell to resolve polyatomic interferences. The ICP-MS is interfaced with an Elemental Scientific Flow Injection Automation System (FIAS). The accuracy of the analysis according to this new method is verified by measuring 2 certified reference materials, BCR 273 and BCR 414. This work presents a number of running parameters, optimised for multi-element analysis of samples with a high TDS sample matrix. This method allows direct measurement of protein samples in their native state: no alteration or digestion is needed, which simplifies the steps for sample preparation. In this study with 6 *E. huxleyi* strains isolated from the environment, our method reveals significant differences between whole cell and intracellular metal quotas for all strains. The intracellular metal composition, interpreted as a truer representation of organisms' metal requirements, shows an environmentally dependent signal. This suggests that, compared with whole cell metal quotas, the metalloproteins are a better indicator of metal requirements of phytoplankton under various environmental conditions.

## Abstract 2

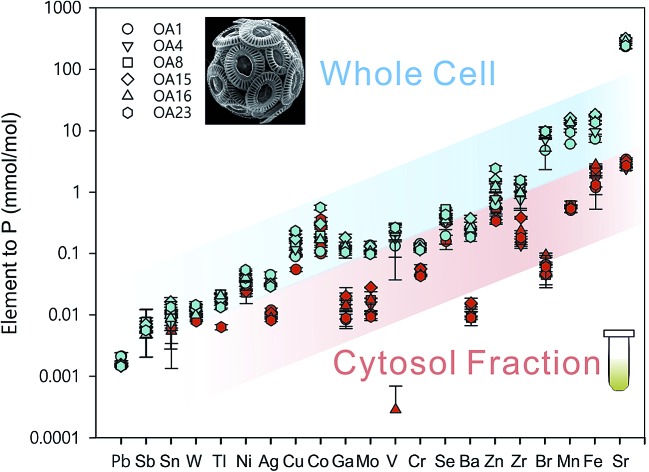
Based on our novel ICP-MS method, we find that the metalloproteins are a better indicator of metal requirements of phytoplankton compared with whole cell metal quotas.

## Introduction

It is difficult to overstate the importance trace metals play in the biogeochemical cycling and productivity of ocean ecosystems; marine phytoplankton rely on micronutrient metals for growth, drive ocean primary productivity, and exert control on the availability of those elements in the environment.^1^ A common tenet of phytoplankton trace-metal research is that the elemental composition of the phytoplankton largely reflects the organisms biological requirement – the cornerstone of this being the largely conserved ratio of macronutrients observed by Alfred Redfield in 1934.^2^ Innumerable studies have explored the Redfield ratio discovering an inherent variability associated with differing community composition,^3^ water chemistry,^4^ temperature,^5^ and many other parameters.^6^ Despite this variability the Redfield ratio of C_106_N_16_P is generally conserved largely due to C, N and P representing significant biochemical pools – carbohydrates rich in C, proteins rich in N, and lipids, DNA, and RNA rich in P-this ratio is rooted in the fundamental structure of life and there may not be sufficient biological flexibility to deviate substantially away from it.^7^

Micronutrient trace metals however account for a far smaller proportion of phytoplankton biomass^8^–^10^ and play a more transient but equally important role in biology as catalytic centres of metalloproteins.^11^ The flexible and substitutable nature of micronutrient trace-metals^12^,^13^ contrasts the relative inflexibility of macronutrient pools that acts to maintain the observed constant ratio.^4^,^7^ Correspondingly, observations of phytoplankton trace-metal quotas have revealed substantially higher variability in their stoichiometry than that of the macronutrients.^8^–^10^,^14^ Alongside high stoichiometric variability, a disconnect between intracellular trace-metal content and interpretable biological requirement has been observed on many occasions^8^,^15^ suggesting an incomplete understanding of the role intracellular metal quotas play in phytoplankton cells. It is well established that whole cell quota does not necessarily represent biological use when it comes to trace-metals, iron storage in ferritin complexes is widespread amongst marine bacteria^16^,^17^ and some eukaryotes,^18^–^20^ increasing evidence of copper storage strategies is emerging in soil bacteria^21^ and many phytoplankton store excess metals in the so called ‘metal-rich granules’ in the cell wall.^22^–^24^ Furthermore, although the expanding numbers and roles of metallic elements implicated in biological functions are striking,^25^ the diversity of biologically functional metal ions is far from well characterised: across the periodic table, only 25 of the 117 elements are known to be essential to all life, with another 7 having possible biological roles for some species.^26^ Therefore, in order to expand our knowledge on the requirements of trace elements by phytoplankton, a method is required to efficiently and accurately quantify the cellular usage of a wide range of elements simultaneously in biological samples.

Here we present a novel method for quantifying the abundance of 32 trace metals simultaneously in both the whole cell digest alongside an operationally defined intracellular fraction. Separation of the intracellular cytosolic fraction from the membrane rich, cell debris fraction provides the foundation towards being able to disentangle intracellular metal presence and biological metal use and allows us to present an extended Redfield ratio for trace metal use.

The method uses inductively coupled plasma-mass spectrometry (ICP-MS), which is highly sensitive for measuring trace elements in a wide variety of sample types and has been employed to analyse the metal compositions of separated proteins.^27^ However, the conventional sample introduction system with ICP-MS requires the total concentration of dissolved solids in samples to be less than 0.2%,^28^ otherwise materials may deposit within the instrument, causing instrument drift and signal suppression.^29^ In order to measure biological samples such as cell lysate or purified proteins, a time-consuming pre-treatment is always needed, which involves pre-concentration, acid or microwave digestion, and dilution with the desired media (*e.g.* 2% HNO_3_). All these steps may introduce unnecessary contamination and uncertainty to the measurement, which will affect the precision of the analysis. Another major drawback of determination by ICP-MS has been polyatomic interferences.^30^ To reduce the impact of polyatomic interferences, modern day quadrupole ICP-MS systems are often equipped with collision/reaction cell technologies. However, the effectiveness of these devices remains a contentious issue, especially when dealing with a complex sample matrix, such as is found in biological materials. These interferences may affect the accuracy of the measurement. Therefore, a robust method to deal with these two major drawbacks is required in order for an accurate determination of metal contents in native proteins.

Some recent studies reported using an aerosol dilution technique as a simple strategy for the analysis of high matrix samples;^31^,^32^ however, they can only measure samples with quite low TDS (less than 3%) and with a relatively large volume (>5 mL sample). For high TDS samples,^33^ developed a method using ICP-MS coupled with an ultra-high matrix introduction system (UHMI) to measure Eu and U in highly saline samples (up to 5 mol L^–1^ NaCl), but such a method requires 100 times on-line dilution for the samples, and therefore is not suitable for analysing elements with low initial concentrations. To date, no systematic strategy for the simultaneous determination of multiple elements in small volume samples with a high TDS matrix by ICP-MS has been reported so far. Therefore, in the present study, we developed and optimised a novel method to satisfy two of our requirements: (i) the capability to measure intracellular elements composition with proteins in their native state (normally containing high TDS to maintain protein conformation), and (ii) the capability to determine a wide range of different elements in small volume cytosol fractions (<1 mL), so that our knowledge of the protein-metal-centres can be expanded. It allows direct analysis of 32 elements simultaneously in samples with a high TDS matrix (up to 30%). The method uses a quadrupole-ICP-MS with a collision/reaction cell to resolve polyatomic interferences.^30^,^34^,^35^ Collision cell technology is operated using kinetic energy discrimination (KED) mode. In this mode, a potential barrier is set between the cell and the quadrupole mass filter. When polyatomic ions pass through a cell pressurized with helium gas, their potential energy is decreased below the KED bias voltage required to enter the quadrupole mass filter, due to the collisions with the inert gas.^36^ The dynamic reaction cell (DRC) approach, removes the interferences through two mechanisms: chemical reactions between the interfering ions and the ammonia gas, and dynamic bandpass tuning (DBT), which precisely controls the bandpass mass filter inside the dynamic reaction cell to exclude the undesired species. The ICP-MS is also interfaced with an Elemental Scientific Flow Injection Automation System (FIAS), which allows direct injection of a micro-volume of sample without the need for offline dilution. The accuracy of the analysis is verified by measuring 2 certified reference materials, BCR 273 (single cell protein) and BCR 414 (plankton).

This work provides valuable parameters that have been optimised for multi-element analysis in a low volume but high TDS sample matrix, and the method allows direct measurement of protein samples in their native state; no alteration is needed, which is time efficient for sample preparation. Such samples can be whole cell lysate or fractions from HPLC separation which contain high percentage concentrations of NaCl, or other high TDS samples such as seawater. We demonstrate an application of the method to determination of an extended Redfield ratio of metal requirements in *E. huxleyi* and determine the difference of this ratio to the extended Redfield ratio of cell quotas developed previously based on whole cell analysis.^9^

## Materials and methods

### Instrument

A PerkinElmer NexION 350D Inductively Coupled Plasma-Mass Spectrometer (ICP-MS) was employed for this study. The ICP-MS is interfaced with an Elemental Scientific Inc. (ESI, USA) PrepFAST M5 Auto-sampler system. The connection of this ancillary equipment to the NexION provides a facility to dilute and also inject micro-volume aliquots of blank, standard and sample solutions directly into the mass spectrometer using the FAST FIAS (Flow Injection Automation System) facility. The injections are measured by the fast-scanning quadrupole of the NexION 350D mass spectrometer and the average cps data is taken from three injections per replicate.

The NexION 350D has a collision/reaction cell to improve signal to background measurements for many elements that suffer from spectral interference. For this study CP grade helium gas (>99.999% purity) was used as a collision cell gas for KED mode (Kinetic Energy Discrimination) and electronic grade ammonia gas (>99.9995% purity) was used as a reactive cell gas, for charge transfer and atom transfer reaction dynamics in DRC mode (Dynamic Reaction Cell). Both cell gas modes are able to be programmed to switch, then stabilise and measure with the ESI FAST FIAS flow injection system.

The ICP-MS was optimised before measurements were made (in standard mode, KED mode, and DRC mode) to maximise sensitivity across the mass range. The sensitivities for Li, Mg, In, Ce, Pb, and U were monitored while ensuring that the CeO^+^/Ce^+^ ratio was maintained at less than 2.5%.

The detailed instrumental settings are listed in Table 1, and parameters used for each analyte are summarised in Table 2. An ESI Pergo Argon Nebulizer Gas Humidifier is also used in this study to reduce salting of the nebuliser.

**Table 1:** Instrumental setting

ICP-MS instrument	NexION 350D
**Plasma condition**	
RF power	1600 W
Plasma gas flow	18 mL min^–1^
Auxiliary gas flow	1.2 mL min^–1^
Nebulizer gas flow	0.8–1.0 mL min^–1^

**Mass spectrometer setting**	
Scanning mode	Peak hopping
Dwell time	Variable, dependent upon FIAS method
Sweeps	1
Readings	50
Replicates	3

**Non-DRC setting**	
RPq	0.25
RPa	0

**Other**	
Loop size	500 μL

**Table 2:** Parameters used in multi-elements analysis

Element	Potential interferences^56^	Mode	Cell gas flow	RPq	Detection limit (ng mL^–1^)
^31^P	^14^N^16^O^1^H, ^15^N^15^N^1^H, ^15^N^16^O, ^14^N^17^O, ^13^C^18^O, ^12^C^18^O^1^H	KED	0.5	0.25	8.67
^77^Se	^40^Ar^37^Cl, ^40^Ca^37^Cl, ^36^Ar^40^Ar^1^H	KED	0.5	0.25	2.6
^7^Li		KED	4.2	0.25	0.42
^24^Mg	^12^C_2_	KED	4.2	0.25	0.92
^27^Al	^12^C^15^N, ^13^C^14^N, ^1^H^12^C^14^N	KED	4.2	0.25	4.09
^47^Ti	^32^S^14^N^1^H, ^30^Si^16^O^1^H, ^32^S^15^N, ^33^N^14^N, ^33^S^14^N, ^15^N^16^O_2_, ^14^N^16^O_2_^1^H, ^12^C^35^Cl, ^31^P^16^O	KED	4.2	0.25	5.46
^55^Mn	^40^Ar^14^N^1^H, ^39^K^16^O, ^37^Cl^18^O, ^40^Ar^15^N, ^38^Ar^17^O, ^36^Ar^18^O^1^H, ^38^Ar^16^O^1^H, ^37^Cl^17^O^1^H, ^23^Na^32^S, ^36^Ar^19^F	KED	4.2	0.25	0.23
^56^Fe	^40^Ar^16^O, ^40^Ca^16^O, ^40^Ar^15^N^1^H, ^38^Ar^18^O, ^38^Ar^17^O^1^H, ^37^Cl^18^O^1^H	KED	4.2	0.25	12.42
^59^Co	^43^Ca^16^O, ^42^Ca^16^O^1^H, ^24^Mg^35^Cl, ^36^Ar^23^Na, ^40^Ar^18^O^1^H, ^40^Ar^19^F	KED	4.2	0.25	0.02
^60^Ni	^44^Ca^16^O, ^23^Na^37^Cl, ^43^Ca^16^O^1^H	KED	4.2	0.25	0.11
^63^Cu	^40^Ar^23^Na	KED	4.2	0.25	0.12
^66^Zn	^50^Ti^16^O, ^34^S^16^O_2_, ^33^S^16^O_2_^1^H, ^32^S^16^O^18^O, ^32^S^17^O_2_, ^33^S^16^O^17^O, ^32^S^34^S, ^33^S_2_	KED	4.2	0.25	2.07
^69^Ga	^35^Cl^16^O^18^O, ^35^Cl^17^O_2_, ^37^Cl^16^O_2_, ^36^Ar^33^S, ^33^S^18^O_2_, ^34^S^17^O^18^O, ^36^S^16^O^17^O, ^33^S^36^S	KED	4.2	0.25	0.001
^74^Ge	^40^Ar^34^S, ^36^Ar^38^Ar, ^37^Cl^37^Cl, ^38^Ar^36^S	KED	4.2	0.25	0.62
^75^As	^40^Ar^35^Cl, ^40^Ca^35^Cl	KED	4.2	0.25	0.04
^88^Sr	^44^Ca_2_	KED	4.2	0.25	0.32
^89^Y		KED	4.2	0.25	0.14
^90^Zr		KED	4.2	0.25	0.05
^98^Mo	^81^Br^17^O, ^41^K_2_O	KED	4.2	0.25	0.09
^107^Ag	^91^Zr^16^O	KED	4.2	0.25	0.06
^111^Cd	^95^Mo^16^O, ^94^Zr^16^O^1^H, ^39^K_2_^16^O_2_^1^H	KED	4.2	0.25	0.66
^118^Sn	^102^Ru^16^O, ^102^Pd^16^O	KED	4.2	0.25	0.58
^121^Sb	^105^Pb^16^O	KED	4.2	0.25	0.21
^138^Ba		KED	4.2	0.25	0.11
^184^W		KED	4.2	0.25	0.04
^205^Tl		KED	4.2	0.25	0.01
^208^Pb	^192^Pt^16^O	KED	4.2	0.25	0.001
^39^K	^38^Ar^1^H	DRC	0.8	0.8	3.34
^44^Ca	^12^C^16^O_2_, ^14^N_2_^16^O, ^28^Si^16^O	DRC	0.8	0.5	45.04
^51^V	^35^Cl^16^O	DRC	0.8	0.8	0.002
^56^Fe	^40^Ar^16^O, ^40^Ca^16^O, ^40^Ar^15^N^1^H, ^38^Ar^18^O, ^38^Ar^17^O^1^H, ^37^Cl^18^O^1^H	DRC	0.8	0.8	0.11
^52^Cr	^40^Ar^12^C, ^35^Cl^16^O^1^H, ^36^Ar^16^O, ^37^Cl^15^N, ^34^S^18^O, ^36^S^16^O, ^38^Ar^14^N, ^36^Ar^15^N^1^H, ^35^Cl^17^O	DRC	0.6	0.8	0.06
^79^Br	^40^Ar^39^K, ^31^P^16^O_3_, ^38^Ar^40^Ar^1^H	DRC	0.6	0.45	0.67

### Reagents

Multi-element calibration standards (10 mg L^–1^) for ICP-MS, including transition metals, high field strength elements, and alkali metals, were purchased from CPA Chem (C.P.A Ltd, Bulgaria). Individual ICP single-element standards (Merck Certipur 1000 mg L^–1^) of P, Y, and Br were also used. All of these calibration standards are traceable to NIST SRM's. The certified materials, BCR 273 and BCR 414, were purchased from Sigma Aldrich. Quartz distilled (QD) acids and 18 MΩ cm H_2_O (Merck Millipore, USA) were used throughout the experiments for sample and standard preparation.

### Sample preparation

All sample preparation steps have been conducted in a trace-metal-clean way. To minimise blank concentrations, all samples and reagent handling prior to trace elements analysis was undertaken in laminar flow hoods in the Clean Laboratory Suite at the Department of Earth Sciences, University of Oxford. A multi-element working standard of 200 μg L^–1^ was prepared by carefully weighing aliquots of multi-elements and individual calibration standards, and diluted with 2% HNO_3_. The working solution was prepared in 50 mL metal-free centrifuge tubes (VWR, USA) that have been immersed in 10% quartz distilled HCl for at least a week before thorough rinsing with 18 MΩ cm water. During the measurement, the working standard was on-line diluted to the desired concentrations by the PrepFAST automated dilution system from the instrument.^37^

The certified materials were weighed carefully with an analytical balance and then completely digested with QD 16 M HNO_3_ + 30% H_2_O_2_ (v/v). The digests were then dried on a hotplate and brought back to the desired volume with 2% HNO_3_.

Intracellular fractions were extracted from six *Emiliania huxleyi* strains isolated from different locations during three research cruises in 2011.^38^ The strains were cultured in Aquil* medium (NCMA) modified from ref. 39 and were kept at 20 °C at 150 μmol photons m^–2^ s^–1^ PAR (photosynthetically active radiation) with a day : night photo-period of 12 : 12 hours. The cells were harvested at the late exponential stage, by centrifugation (4100 rpm for 20 min) and rinsed 3 times with chelex-cleaned synthetic ocean water^40^ to remove the weakly bound surface metals. The cell pellets were then resuspended in an extraction buffer (20 mM Tris-Cl, pH 8.0)^41^ and sonicated 6 times for 30 s bursts (on ice) with 1 min interval between sonication (Hielscher UP200S ultrasonic processor, 70% amplitude).^42^ The cell lysate was centrifuged at 1500*g* at 4 °C for 30 min, and the supernatant containing soluble content of the cytoplasm (S1) was passed through a pre-cleaned 0.22 μm PTFE membrane before being subjected to trace element analysis by ICP-MS. Protein concentrations in S1 were determined *via* Bradford assay.^43^,^44^ Two aliquots of S1 fraction were taken from each sample: one for direct element analysis, and the other was digested using the same method for digesting the certified materials. The pellet (P1), containing most insoluble membrane fractions, was kept in a 100 °C water bath for 2 minutes and then digested with 0.5 mol L^–1^ NaOH at 70 °C for 60 minutes. We then centrifuged the digest at 10 000*g* for 10 minutes to get the supernatant (S2) containing the cellular debris and the pellet (P2) containing the metal-rich granules (MRG)^24^ and the coccoliths. These two subcellular fractions were then acid digested as previously described and the metal compositions were also determined by the ICP-MS.

## Results and discussion

### KED and DRC comparison

In order to optimise the method for multi-element measurement for protein samples from different *E. huxleyi* strains, each element was run in both KED and DRC mode, and the standard curves obtained were then compared from these two different modes. Raw counts and signal/noise ratios were also taken into consideration when comparing the methods. It is optimal to have both high counts and high signal/noise ratio for the measurement. We found that these two requirements can be achieved for most of the elements under KED mode. With a complex sample matrix, if the elements can be measured in the collision mode, it is preferable to avoid the reaction mode. The use of reactive gases, such as ammonia gas, is reported to be limited due to clustering reactions.^45^,^46^ If adequate steps are not taken to control the appearance of cluster ions, undesired products may be formed, such as M(NH_3_)_*n*_^+^ clusters, M(NH)(NH_3_)_*n*_^+^, or M(NH_2_)(NH_3_)_*n*_^+^.^47^,^48^ Therefore, it is crucial to optimise the bandpass setting on the quadrupole inside the dynamic reaction cell if using the DRC mode, to avoid the interferences originating from the cluster ions depending on the sample matrix.

The elements that benefit from being analysed using KED mode are listed in Table 2. The cell gas flow in the KED mode was 4.2 mL min^–1^ for most of the elements (Table 2). However, due to the low ionisation efficiency and the loss of energy from collision with helium gas, the signals for P and Se are very low with such a high cell gas flow. Therefore, a cell gas flow rate of 0.5 mL min^–1^ was employed to measure these elements (Table 2).

We note that KED mode was not ideal for every element; collision with helium gas not only suppresses signals of polyatomic interferences, but also significantly suppresses the analyte signal. This could lead to a low signal/noise ratio, which affects the accuracy of the measurement. Therefore, for elements such as K and Br, DRC mode was selected as a better option.

### DRC optimisation

In the dynamic reaction cell, the interfering species were eliminated by reaction with NH_3_ gas, leading to the form of new polyatomic products that do not interfere with the analytes of interest. The reactions are between ions that are at thermodynamic equilibrium, which requires a relatively high pressure inside the reaction cell and a relatively low energy of the ions.^49^ Therefore, predictable exothermic reactions would take place within the cell. However, not all spectral interferences have an exothermic reaction with the reactive gas, in which case the interferences cannot be removed by the reaction cell.^36^ Furthermore, the reactive gas may also create new polyatomic interferences at the mass of the analytes of interest.^50^,^51^ To suppress the interferences from these undesired cluster ions, the ammonia gas flow and the bandpass tuning parameter value need to be optimised.

**Fig. 1:**
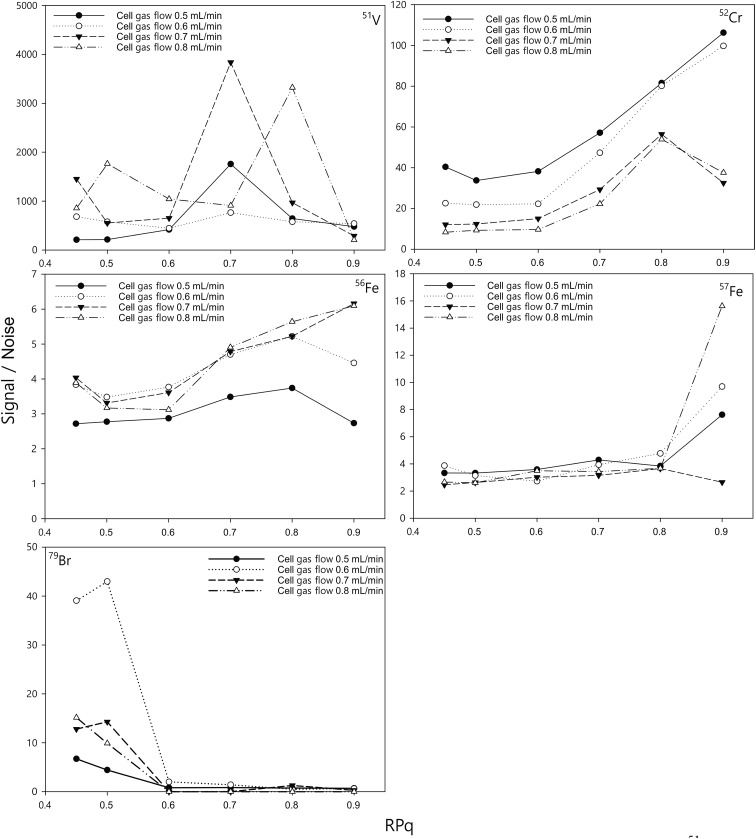
Effect of the ammonia reaction gas flow rate and RPq on the signal/noise of ^51^V, ^52^Cr, ^56^Fe, ^57^Fe, and ^79^Br. The concentration of the standards was 10 ng mL^–1^ and the blank was 2% HNO_3_.

Here, we assess and optimise the gas flow rate and the bandpass tuning parameter (RPq) for different elements by measuring a 10 ng mL^–1^ standard solution and comparing the signals with those from a blank measurement of 2% HNO_3_ (Fig. 1). The optimal cell gas flow and RPq values varied for different elements (Table 3). With a certain RPq value, higher ammonia gas flow rates resulted in lower signals for the elements, but not necessarily a lower signal to noise ratio (S/N). Similarly, with a certain gas flow rate, a higher RPq value also resulted in lower signals for the measurement, but a higher signal to noise ratio for many elements, such as Cr and Fe. It has to be noted that, the optimum RPq values and cell gas flow rates were not purely dependent on the signal to noise ratio. The raw signals for the elements are also important. If the raw signals were too low, such as when the RPq value was set to 0.9, the precision of the measurements would be significantly affected. Therefore, the raw counts also need to be considered when optimising the method. With the instrument in this study, we allowed a period of 30 seconds to change between different cell modes. Therefore, considering the efficiency of the measurement for each sample, two cell gas flows were chosen (0.6 mL min^–1^ and 0.8 mL min^–1^) to achieve a relative high S/N for all elements, and the RPq values employed are listed in Table 2.

**Table 3:** Ca interferences on the measurements for Fe and Sr. The data were obtained by measuring pure Ca standards at 0.8 mL min^–1^ cell gas flow rate and 0.8 RPq value. The data are reported here as the net intensity (counts of the analyte/counts of the internal standard) and BEC (Background equivalent concentration). —: below detection limit

	^56^Fe (KED)	BEC (ng mL^–1^)	^57^Fe (KED)	BEC (ng mL^–1^)	^56^Fe (DRC)	BEC (ng mL^–1^)	^57^Fe (DRC)	BEC (ng mL^–1^)	^88^Sr (KED)	BEC (ng mL^–1^)
Blank	0.248	—	9.98 × 10^–4^	—	0.061	—	0.003	—	6.32 × 10^–4^	—
100 ng mL^–1^ Ca	0.270	—	1.16 × 10^–3^	—	0.062	—	0.004	—	4.52 × 10^–4^	—
200 ng mL^–1^ Ca	0.269	—	1.04 × 10^–3^	—	0.064	—	0.006	0.467	9.83 × 10^–4^	—
500 ng mL^–1^ Ca	0.270	—	8.48 × 10^–4^	—	0.064	—	0.007	0.876	6.74 × 10^–4^	—
1 μg mL^–1^ Ca	0.250	—	8.32 × 10^–4^	—	0.065	—	0.022	6.295	1.33 × 10^–3^	0.172
2 μg mL^–1^ Ca	0.251	—	9.44 × 10^–4^	—	0.077	0.095	0.039	12.744	1.58 × 10^–3^	0.233
5 μg mL^–1^ Ca	0.253	—	8.46 × 10^–4^	—	0.100	0.33	0.092	32.687	3.94 × 10^–3^	0.813
10 μg mL^–1^ Ca	0.238	—	9.78 × 10^–4^	—	0.136	0.69	0.163	59.146	8.26 × 10^–3^	1.87

### Ca interferences

**Fig. 2:**
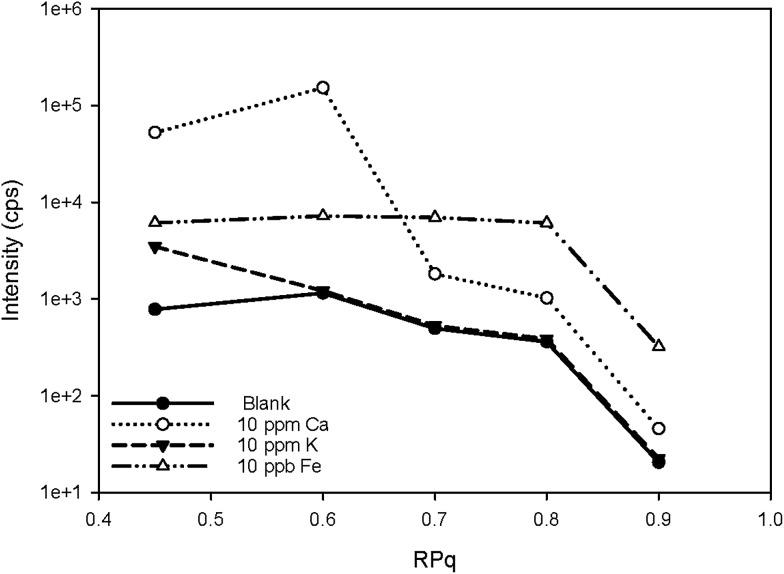
Signal counts of ^56^Fe and potential spectral overlap cluster ions at 56 as a function of RPq. The ammonia gas flow rate was 0.8 mL min^–1^.

**Fig. 3:**
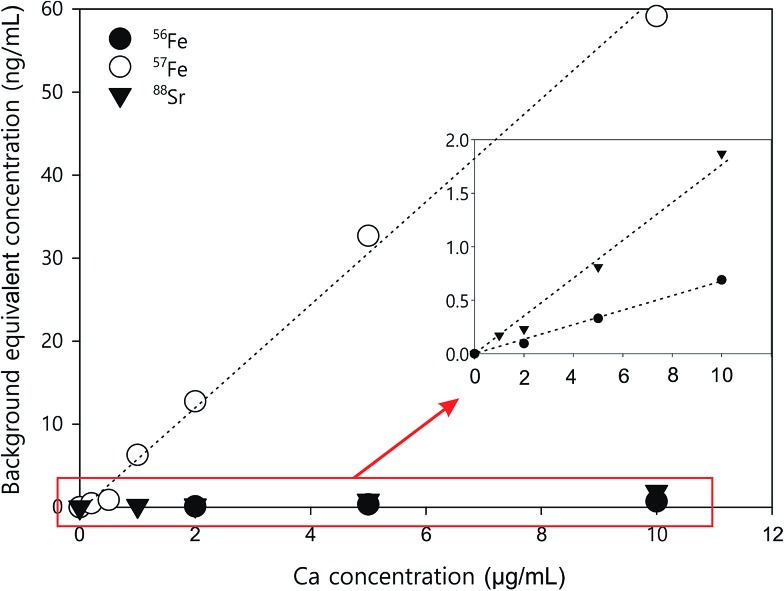
Ca standard curves for correcting background equivalent concentrations for ^88^Sr (KED mode), ^56^Fe (DRC mode), and ^57^Fe (DRC mode).

With the optimisation above, most of the interferences are largely removed, especially those originating from argon clusters. However, there may still be some polyatomics that are harder to eliminate, such as ^40^Ca^16^O^+^, and ^40^Ca^14^N^1^H_2_^+^ that interfere with ^56^Fe. Calcium is very abundant in the environment, estimated at about 15 mg L^–1^ in average river water,^52^ and above 400 mg L^–1^ in seawater.^53^ The calcium concentration is also expected to be high in digests of calcifying organisms, such as coral and the *E. huxleyi* strains in this study.^38^ The potential interference with high calcium concentrations were studied by introducing Ca standard solutions (from 100 ng mL^–1^ to 10 μg mL^–1^) into the ICP-MS and measuring the concentration of different elements with the optimised method. With the exception of Sr and Fe, there was no observable elevation of signals from blank level for all other elements in this study. The blank was examined by introducing 2% HNO_3_. The net intensities and the background equivalent concentrations caused by Ca interferences were listed in Table 3. The net intensity was calculated as follows:
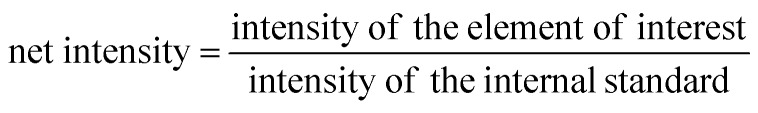


Two iron isotopes, ^56^Fe and ^57^Fe, were monitored simultaneously in both KED and DRC mode. ^56^Fe is the most abundant Fe isotope (91.7%), but it has interferences from the most abundant argon oxide, ^40^Ar^16^O^+^. To avoid this interference, ^57^Fe may be measured instead. However, the isotopic abundance of naturally occurring ^57^Fe is only 2.2%, and it also has interferences originating from ^40^Ar^17^O and ^40^Ar^16^O^1^H, which may be too high to neglect. Under most conditions, it is better to eliminate these interferences in DRC mode, because the intensity of Fe would not be suppressed as much as in KED mode. Further the noise from the interferences was much lower, which means a much higher signal to noise ratio in the DRC mode. However, with Ca-rich samples, measurements in the DRC mode may still be problematic. As shown in Table 2, in the DRC mode, although low Ca concentration (less than 1 μg mL^–1^) did not have a significant impact on the signal for ^56^Fe, higher Ca concentration may significantly increase the background equivalent concentration of ^56^Fe. For ^57^Fe in the DRC mode, Ca has an even greater impact, which may be due to the formation of ^40^Ca^14^N^1^H_3_ from the reaction between Ca and the ammonia gas. Therefore, we conducted a further optimisation with the DRC method for ^56^Fe. A 10 μg mL^–1^ Ca standard was measured as ^56^Fe at the optimised cell gas flow rate (0.8 mL min^–1^) with various RPq values. A 10 ng mL^–1^ Fe standard and a blank solution (2% HNO_3_) were also measured under the same condition. The data was plotted and compared in Fig. 2. With the same cell gas flow rate, the intensity from the Ca interference was the highest when the RPq value was set to 0.6, and the equivalent intensity decreased with higher RPq values. However, the background equivalent intensity from Ca interference cannot be fully eliminated even with the highest RPq value (RPq = 0.9, Fig. 2). The signals from the Fe standard stayed constant between an RPq value of 0.45 to 0.8, and the intensity decreased significantly when the RPq value was 0.9. The difference between Fe signals and the Ca interference was largest when the RPq was 0.8. The potential interference of ^39^K^14^N^1^H_3_^+^ was also investigated by introducing a 10 μg mL^–1^ K standard solution and measured with various RPq values. It is obvious that the signals from a K interference were suppressed to blank level when the RPq value was higher than 0.6. Therefore, 0.8 is the optimised RPq value for measuring ^56^Fe in the DRC mode, and the background equivalent concentration from 10 μg mL^–1^ Ca was less than 1 ng mL^–1^ (Fig. 3 and Table 3). This is acceptable when the Fe concentration in samples is higher than 5 ng mL^–1^, and Ca concentration is less than 10 μg mL^–1^. When Ca concentration is higher than 10 μg mL^–1^, a separate calibration with Ca standards can be performed to take the interference into account with the Fe measurement. However, if the Ca concentration is too high, such as 400 μg mL^–1^ in seawater, the interference may be too large to allow a precise measurement of Fe in DRC mode.

In KED mode, the introduction of a Ca solution did not affect the signals for either ^56^Fe or ^57^Fe. From 100 ng mL^–1^ to 10 μg mL^–1^, the signals from Ca standards stayed constant at the background level. However, as mentioned before, there was interference from argon oxides for both ^56^Fe and ^57^Fe, and the signal to noise ratio for Fe measurement was not high. If the iron concentration in samples is too low (less than 5 ng mL^–1^), it is challenging to get an accurate measurement in this mode.

Other than iron, calcium can also interfere with the measurement of ^88^Sr in KED mode. As shown in Table 3, when Ca concentration was larger than 1 μg mL^–1^, there was a noticeable increase of the ^88^Sr signal. This may be due to impurity in the Ca standard. The Sr concentration is about 0.4 ng mL^–1^ in the Ca standard used in this study. The ^44^Ca_2_^+^ interference for ^88^Sr should be relatively low, as the natural abundance of ^44^Ca (2.09%) is much lower than ^40^Ca (96.94%). Nonetheless, the natural abundance of the other stable isotopes of Sr, ^84^Sr (0.56%), ^86^Sr (9.86%), and ^87^Sr (7%), is much lower than ^88^Sr (82.58%). At low ng mL^–1^ levels, the intensity of those isotopes from ICP-MS measurement is too low to be reliable. Therefore, ^88^Sr is still the most preferable Sr isotope for analysis; however care may be needed when measuring it in Ca-rich samples. If the Ca concentration in samples is higher than 10 μg mL^–1^, a separate Ca calibration should be performed to measure the Ca interference with the analysis. The impurity in the standards should also be taken into consideration.

### Method validation with reference materials

In order to verify the proposed method, two certified reference materials BCR 273 and BCR 414 were analysed, and the results are shown in Table 4. BCR 273 is a single cell protein powder of bacterial origin, and BCR 414 is a plankton powder. They are both biological materials which likely have a similar sample matrix with the *E. huxleyi* samples in this study. As shown in Table 4, the recovery of the measurement was very high (close to 100%) for most elements in the list, including all the elements that have certified values. In order to match the range of concentrations of different elements in protein samples extracted from *E. huxleyi* in this study, only about 1 mg of the reference materials were weighed and digested for the measurement. Therefore, the concentration of some of the elements (*e.g.* Se, Sn and Tl) were too low to be measured accurately, resulting in a low recovery for those elements. Nonetheless, in general, the method is good for measuring the elements proposed in biological sample matrix. The precision and accuracy of the measurement was also monitored by repeatedly measuring a 5 ng mL^–1^ quality-control standard solution (CPA Chem, C.P.A. Ltd, Bulgaria) along with every 5 to 10 samples. The relative standard deviation of the measurement is normally less than 3%.

**Table 4:** Analytical results and certified values for various elements in standard samples^*a*^

Certified materials	Element	Unit	Certified value	Measured value	Recovery (%)
BCR 414: plankton	As	μg g^–1^	6.82 ± 0.28	6.78 ± 0.24	99 ± 3
	Cd	μg g^–1^	0.383 ± 0.014	0.385 ± 0.017	100 ± 4
	Cr	μg g^–1^	23.8 ± 1.2	23.3 ± 0.5	98 ± 2
	Cu	μg g^–1^	29.5 ± 1.3	30.7 ± 2.9	104 ± 10
	Mn	μg g^–1^	299 ± 12	290 ± 17.7	97 ± 6
	Ni	μg g^–1^	18.8 ± 0.8	18.8 ± 1.0	100 ± 5
	Pb	μg g^–1^	3.97 ± 0.19	3.78 ± 0.35	95 ± 9
	Se	μg g^–1^	1.75 ± 0.10	1.27 ± 0.7	73 ± 40
	V	μg g^–1^	8.10 ± 0.18	7.3 ± 0.68	90 ± 8
	Zn	μg g^–1^	112 ± 3	103.03 ± 7.5	92 ± 7
	Co*	μg g^–1^	1.43 ± 0.06	1.46 ± 0.21	102 ± 15
	K*	μg g^–1^	7.55 ± 0.17	7.33 ± 0.72	97 ± 10
	Fe*	mg g^–1^	1.85 ± 0.19	1.90 ± 0.13	103 ± 7
	Mo*	μg g^–1^	1.35 ± 0.20	1.54 ± 0.22	114 ± 16
	Sr*	μg g^–1^	261 ± 25	254 ± 20	97 ± 8
	Al**	mg g^–1^	1.80 ± 0.03	1.85 ± 0.16	103 ± 9
	Ba**	μg g^–1^	31 ± 2	26.17 ± 2.43	84 ± 8
	Br**	μg g^–1^	55 ± 1	33.90 ± 3.38	62 ± 6
	Ca**	mg g^–1^	65 ± 2	74.5 ± 7.8	115 ± 12
	Mg**	mg g^–1^	2.4 ± 0.08	2.48 ± 0.19	103 ± 8
	P**	mg g^–1^	12.3 ± 0.6	13.9 ± 1.07	113 ± 8
	Sn**	μg g^–1^	1.18 ± 0.12	0.40 ± 0.14	34 ± 12
	Tl**	μg g^–1^	0.047 ± 0.002	0.022 ± 0.006	47 ± 13
	Ti**	mg g^–1^	48 ± 5	45.62 ± 12.53	95 ± 26
BCR 273: single cell protein	Ca	mg g^–1^	11.97 ± 0.14	11.02 ± 0.63	92 ± 5
	K	mg g^–1^	2.22 ± 0.05	2.06 ± 0.09	93 ± 4
	P	mg g^–1^	26.8 ± 0.4	26.8 ± 0.4	100 ± 1
	Fe	mg g^–1^	0.156 ± 0.004	0.143 ± 0.007	92 ± 4
	Mg*	mg g^–1^	2.72 ± 0.11	2.72 ± 0.11	100 ± 4

^a^*: indicative values. **: values for information only.

### Elements in intracellular fractions from different *E. huxleyi* strains

The method is applied to measuring intracellular extracts from 6 *E. huxleyi* strains. Whilst our cellular extract fraction is operationally defined and likely contains the majority of the cells cytoplasimic and soluble material, a large proportion of the metals contained in this fraction are suspected to originate from metalloproteins.

**Fig. 4:**
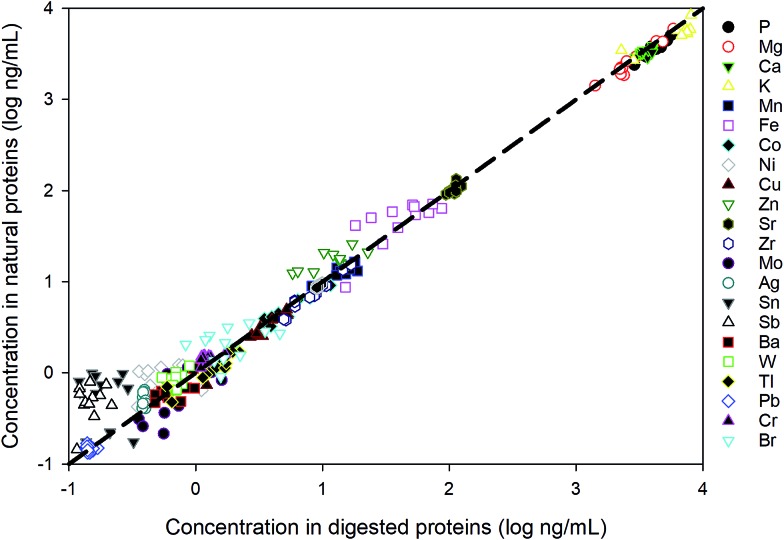
Comparison between direct measurement of proteins in the native state and the measurement after acid digestion.

The strains were cultured in duplicates and 12 samples were extracted. To evaluate the performance for the direct measurement, duplicate aliquots were taken from each sample for the ICP-MS analysis: one aliquot was directly diluted in the extraction buffer (20 mM Tris, pH 8.0) and the other has been acid digested in the same way as for the reference materials. As shown in Fig. 4, most values measured from digested proteins are comparable with those obtained by directly measuring the native proteins, indicating that the pre-treatment for these samples are unnecessary and this method is robust in measuring dissolved native proteins. The elements not listed in the figure are the ones that fall below the detection limits, suggesting an extremely low abundance of those elements in the proteins of *E. huxleyi*.

The intracellular element compositions are listed in Table 5. The data are normalised to protein concentrations for easy comparison between strains. The variations between different *E. huxleyi* strains are negligible for some elements, such as Mo, Br and Tl. However, for other elements, there are significant differences between different strains (*p* < 0.01). An example is given in Table 6, in which the significance of Cu variation (normalised to protein concentration) between different isolated *E. huxleyi* strains are analysed by *t*-test, and the *p* values are labelled with different colours. Interestingly, the difference in Cu concentrations is insignificant (*p* > 0.05, green in Table 6) between strains isolated from the same environment: both OA1 and OA4 were isolated from North Sea, whilst OA15 and OA16 were isolated from the Southern Ocean. Intracellular Cu concentrations are generally higher in strains from the Southern Ocean than those from the North Sea. However, although Cu concentration data are not available from the exact location from which the strains were isolated, dissolve Cu concentrations are generally higher in the North Sea than in the Southern Ocean (Fig. S1†). Such an inverse relationship between intracellular Cu concentration and environmental concentration may arise because the strains from low Cu area have a better ability to acquire Cu, produce Cu-binding proteins, or store Cu intracellularly, since there is much more Cu in the laboratory growth media than in the natural environment (Table S1†). Nevertheless, the difference in intracellular Cu concentrations between strains from different environments are much more significant (*p* < 0.05, orange; *p* < 0.01, red), despite the fact that all the strains have been maintained under the same lab conditions for more than 6 years. This trend generally prevails across most elements analysed including Fe and Mn, whist in whole cell, this trend is not as significant. This suggests that the intracellular metal composition (metal content in metalloproteins) in these strains may be an indicator of the metal requirements of these strains that have evolved under different environmental chemistries. Nonetheless, these data demonstrate that the method is sufficiently sensitive to identify interstrain differences.

**Table 5:** Composition of different elements in proteins extracted from *E. huxleyi* strains. The unit for P, Mg, Ca, and K is μg per mg protein, and for all other elements is ng per mg protein. Data are presented as average value ± standard deviation between biological replicates, *n* = 4

	OA1	OA4	OA8	OA15	OA16	OA23
^31^P	19.2 ± 2.5	16.4 ± 1.6	16.4 ± 0.9	16.6 ± 1.8	14.5 ± 1.7	59.6 ± 4.9
^24^Mg	14.6 ± 5.6	9.1 ± 2.7	15.3 ± 6.9	22.6 ± 5.5	7.6 ± 0. 6	25.8 ± 1.4
^55^Mn	58.9 ± 3.2	59.1 ± 11.6	67.5 ± 7.5	57.7 ± 6.3	51.6 ± 7.3	149.3 ± 26.4
^56^Fe	143 ± 86	152 ± 30	248 ± 49	251 ± 39	248 ± 18	313 ± 22
^59^Co	12.5 ± 1.4	16.2 ± 2.4	18.5 ± 0.9	29.0 ± 2.2	13.3 ± 1.1	108 ± 16
^60^Ni	3.11 ± 0.87	3.37 ± 1.00	2.85 ± 1.34	2.71 ± 1.22	3.06 ± 1.25	9.37 ± 3.46
^63^Cu	7.4 ± 1.7	9.9 ± 1.2	13.0 ± 0.5	17.1 ± 1.3	17.4 ± 1.5	31.2 ± 2.9
^66^Zn	49 ± 19	55 ± 14	71 ± 23	93 ± 18	55 ± 6	110 ± 35
^88^Sr	636 ± 9	358 ± 41	466 ± 61	518 ± 74	350 ± 22	1189 ± 69
^90^Zr	29.1 ± 5.0	21.0 ± 3.1	31.8 ± 3.7	63.7 ± 3.1	34.2 ± 3.1	82.2 ± 8.9
^98^Mo	2.14 ± 0.38	2.42 ± 0.91	3.47 ± 0.62	5.10 ± 1.42	2.80 ± 1.10	4.66 ± 1.88
^107^Ag	2.74 ± 0.21	1.82 ± 0.51	2.15 ± 0.47	2.14 ± 0.58	1.55 ± 0.18	4.45 ± 0.52
^118^Sn	2.36 ± 1.30	1.48 ± 1.14	2.70 ± 2.07	2.10 ± 1.57	1.06 ± 0.73	3.74 ± 4.02
^121^Sb	1.92 ± 2.04	1.08 ± 0.58	1.89 ± 1.22	1.62 ± 1.31	0.97 ± 0.58	3.50 ± 2.18
^138^Ba	4.24 ± 0.86	2.36 ± 0.29	2.52 ± 0.22	4.04 ± 1.06	2.30 ± 0.42	6.28 ± 1.17
^184^W	4.92 ± 0.81	3.33 ± 0.46	3.70 ± 0.85	3.22 ± 0.74	2.41 ± 0.12	7.36 ± 0.51
^205^Tl	6.86 ± 1.15	6.66 ± 0.92	7.19 ± 0.59	5.79 ± 1.11	5.43 ± 0.75	6.59 ± 0.94
^208^Pb	0.95 ± 0.08	0.53 ± 0.04	0.66 ± 0.10	0.62 ± 0.05	0.51 ± 0.02	1.57 ± 0.08
^39^K	18.5 ± 3.6	24.0 ± 4.0	42.5 ± 8.4	27.4 ± 5.8	25.8 ± 4.0	64.8 ± 8.5
^44^Ca	105 ± 49	45 ± 28	57 ± 35	59 ± 37	55 ± 24	161 ± 95
^52^Cr	6.26 ± 0.82	3.90 ± 0.64	4.72 ± 0.84	5.45 ± 1.27	3.76 ± 0.30	11.24 ± 2.01
^79^Br	11.2 ± 2.0	7.9 ± 2.9	7.3 ± 3.9	6.7 ± 2.9	12.0 ± 3.2	24 ± 12

**Table 6:** Variations of Cu concentrations in proteins (upper-table) and in whole cell digest (lower-table) from different strains (*p* value resulted from *t*-test between different strains, and labelled with colours. Green: *p* > 0.05; orange: *p* < 0.05; red: *p* < 0.01). The locations of strains isolated are in brackets. NS: North Sea; BB: Bay of Biscay; SO: Southern ocean; GS: Greenland Sea. The locations were reported in ref. 38

Cu (intracellular)	OA1 (NS)	OA4 (NS)	OA8 (BB)	OA15 (SO)	OA16 (SO)	OA23 (GS)
OA1 (NS)	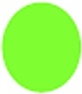	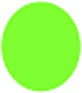	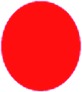	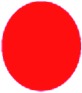	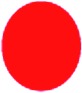	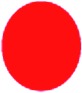
OA4 (NS)	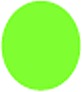	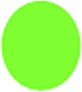	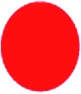	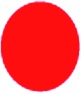	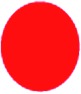	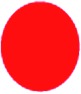
OA8 (BB)	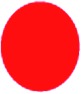	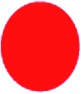	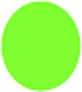	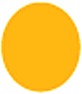	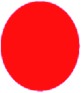	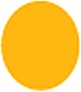
OA15 (SO)	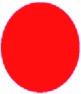	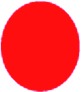	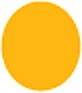	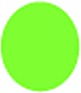	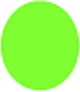	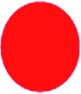
OA16 (SO)	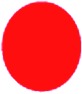	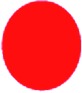	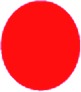	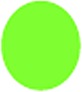	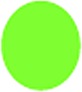	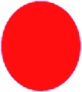
OA23 (GS)	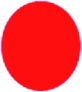	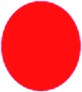	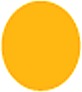	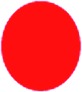	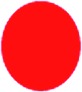	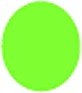

Cu (whole cell)	OA1 (NS)	OA4 (NS)	OA8 (BB)	OA15 (SO)	OA16 (SO)	OA23 (GS)
OA1 (NS)	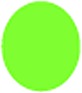	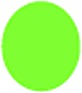	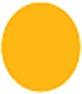	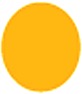	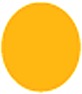	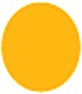
OA4 (NS)	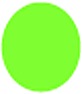	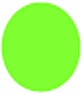	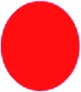	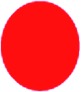	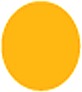	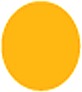
OA8 (BB)	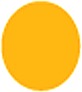	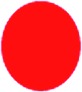	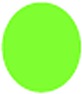	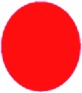	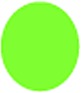	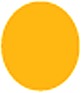
OA15 (SO)	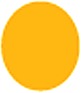	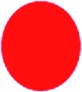	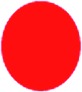	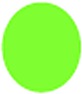	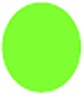	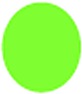
OA16 (SO)	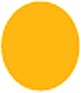	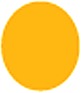	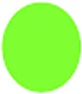	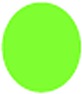	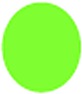	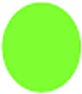
OA23 (GS)	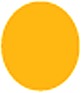	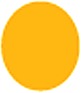	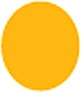	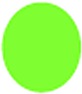	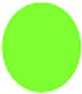	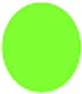

### Whole cell metal quota and intracellular metal requirement

**Fig. 5:**
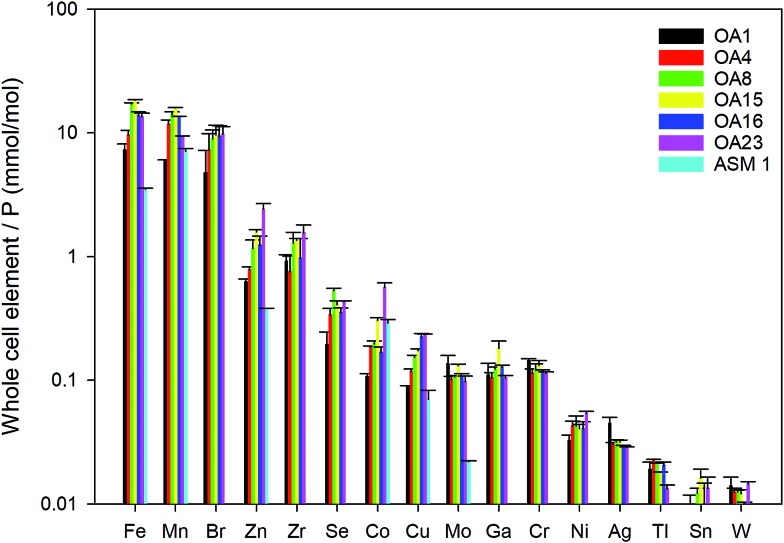
Whole cell metal-quota in different *E. huxleyi* strains (normalised to P). Data for OA strains are from this study and data for the strain ASM1 are from ref. 9.

**Fig. 6:**
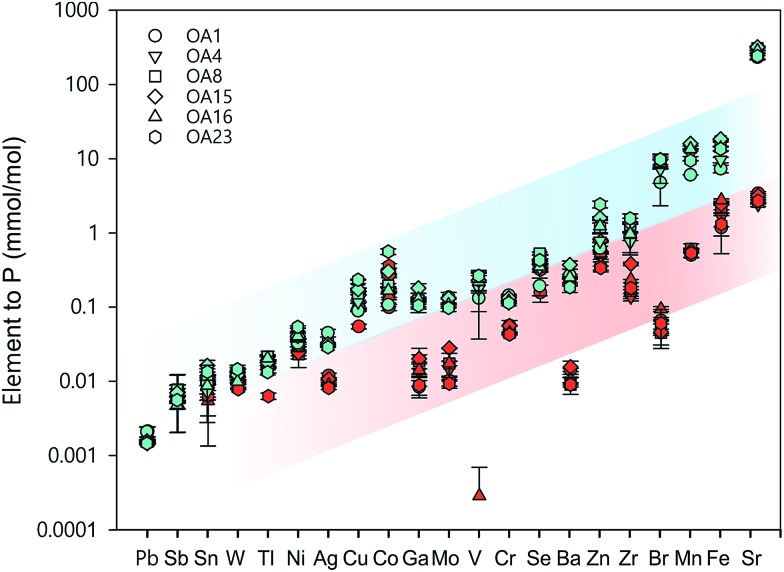
Comparison of whole-cell metal quota (blue symbols, normalised to phosphorus concentration in the whole cell) with total intracellular metal quota (red symbols, normalised to phosphorus concentration in the whole cell) for different *E. huxleyi* strains.

Whole cell metal quota was also determined in this study for those different *E. huxleyi* strains (Fig. 5). The bulk whole cell metal quotas are variable, with OA 23 (from Greenland Sea) having a significant higher Co and Zn concentration, and OA 15 and 16 (both from Southern Ocean) having the highest Fe concentrations. These strains may have evolved to have a better strategy to transport or store the elements that were limited in the environment from which they were isolated. The range of concentrations for the essential trace elements in these whole cell digests such as Fe, Mn, Cu, Zn, and Mo, are comparable with what has been reported in previous studies (Fig. 5),^9^,^10^,^54^,^55^ suggesting the method used in this study is reliable in determining these elements. Other than that, our new method affords simultaneous determination of a wider range of elements than many previous studies. Therefore, this study significantly expands our knowledge of element compositions at the whole cell level and provides a good potential to discover some proteins that bind with some usual metals, such as Pb, Sb, and W (Fig. 6). Based on the data from this study, we worked out an extended Redfield ratio for *E. huxleyi* strains (omitting C, N, and S):

(P_1_K_0.47_Mg_0.35_Ca_124_)_1000_

Sr_287_Fe_11.9_Mn_11.1_Zn_1.27_Cu_0.15_Co_0.26_Mo_0.1_

**Ni**_**0.42**_ **Ga**_**0.12**_ **Zr**_**1.14**_ **Ag**_**0.03**_ **Sn**_**0.01**_ **Ba**_**0.25**_ **W**_**0.01**_ **Tl**_**0.02**_ **V**_**0.2**_ **Cr**_**0.12**_

This formula is comparable to that from ref. 9 for *E. huxleyi*, which is shown below:

(P_1_K_0.84_Mg_0.13_Ca_142_)_1000_

Sr_336_Fe_3.5_Mn_7.1_Zn_0.38_Cu_0.07_Co_0.29_Cd_0.36_Mo_0.022_

The quotas in both of formulas are within the same order of magnitude, and the differences are due to (1) the likely differences between strains isolated from different parts of the ocean, and (2) the differences between the two growth media, for example, our media has higher concentrations of Fe and Mn than ref. 9, but no addition of Cd (Table S1, ESI materials†).

In Fig. 6, the whole cell metal quota and the total intracellular metal quota (excluding the membrane fraction) of the *E. huxleyi* strains are directly compared. There are overlaps with these concentrations for some elements (*e.g*., Ni and Co), suggesting those elements that are high in the intracellular fractions, are most likely to bind with metalloproteins. While for other elements such as Fe and Mn, there were large differences between the whole cell and intracellular metal concentrations, indicating that these elements may be largely stored in the membrane fractions (*e.g.*, metal-rich granules).^24^ The quotas of the extracted cytosol fractions are shown as follows:

(P_1_K_0.36_Mg_0.28_Ca_0.2_)_1000_

Sr_2.7_Fe_1.8_Mn_0.56_Zn_0.49_Cu_0.11_Co_0.19_Mo_0.06_

**Ni**_**0.027**_ **Ga**_**0.013**_ **Zr**_**0.21**_ **Ag**_**0.01**_ **Sn**_**0.008**_ **Ba**_**0.011**_ **W**_**0.01**_ **Tl**_**0.015**_ **V**_**0.0005**_ **Cr**_**0.047**_

The quotas for bio-essential elements in this formula are of the same order of magnitude to those found by ref. 9 for average cell quotas for 16 different species of phytoplankton:

(P_1_K_1.7_Mg_0.56_Ca_0.5_)_1000_

Sr_5.0_Fe_7.5_Zn_0.8_Cu_0.38_Co_0.19_Mo_0.03_

Compared to the whole cell element quota, the relative importance of some elements is greater in the cytosol fractions. For instance, Mn/Co in whole cell quotas is about 43, compared to a ratio in the cytosol fraction of about 3. The cellular requirements of elements such as Co may be under estimated by analysis of cell quotas alone. Furthermore, using the method in this study, we are able to measure many other elements that were overlooked in previous studies. The elements not present in ref. 9 are highlighted in bold above. The biological roles for those elements are currently unclear. It may be that those elements are imported mistakenly into cells without any essential role for biology, but it is also possible that those elements are essential for cells at trace levels. Further studies are needed to establish if specific metalloproteins produced by *E. huxleyi* require those elements as metal centres.

Nonetheless, it is clear that the whole cell element quotas are not equivalent to element requirements by cells. Phytoplankton cells may take up some elements but do not necessarily make use of them.^42^ Therefore, more work should be done in the future to investigate the element usage and storage strategy by phytoplankton under various environmental conditions and to further constrain the metal requirements of the phytoplankton to understand the chemical limitations on oceanic productivity in the past and future.

## Conclusions

We developed and optimised a method for measuring 32 elements simultaneously in low volumes of high TDS matrix sample in 10 min. The method allows direct measurement of these samples with no further handling, which simplifies the sample preparation. Therefore the method has great potential to be used daily for screening multi-elements with a high throughput of high TDS/matrix samples in laboratories performing trace metal, bio-clinical, and toxicology studies. The combination of minimal sample manipulation, small sample volume, and high sample throughput suggest the method could be particularly applicable for the analysis of blood serum or plasma samples from large cohort bio-clinical studies where both accuracy of analysis and minimal sample volume are critically important. The accuracy of the analysis is verified by measuring 2 certified reference materials, BCR 273 and BCR 414. The long-term reproducibility of the method is verified by repeated measurement of a quality-control multi-element standard solution from CPA Chem and digested BCR 414 alongside each batch of samples. The method has then been used to determine the trace element composition of six *E. huxleyi* strains isolated from different locations. Both the whole cell and intracellular metal compositions of these 6 *E. huxleyi* strains were analysed. There are significant differences between the whole cell and the intracellular metal quotas for all strains, and the intracellular metal composition shows a strong environmentally dependent signal. This suggests that, comparing with whole cell metal quotas, the metalloproteins may be a better indicator of the true metal requirements of phytoplankton evolved under various environmental conditions.

## Conflicts of interest

There are no conflicts to declare.

## Supplementary Material

Supplementary information
