# Supplementary material for: Direct measurement of multi-elements in high matrix samples with a flow injection ICP-MS: application to the extended Emiliania huxleyi Redfield ratio
Source: J Anal At Spectrom. 2018 May 29;33(7):1196–208. doi: 10.1039/c8ja00031j (PMC6032269; doi:10.1039/c8ja00031j)
Supplement: Supplementary file 1 [file JA-033-C8JA00031J-s001.pdf]

Electronic Supplementary Material (ESI) for Journal of Analytical Atomic Spectrometry.
This journal is © The Royal Society of Chemistry 2018

Table S1 Composition of different trace elements in growth media.

Component	Molar concentration in medium	Concentration (*Ho et al., 2003*)
Fe	1.00×10^{-6}M	4.5×10^{-7}M
Zn	7.97×10^{-8}M	8.0×10^{-8}M
Mn	1.21×10^{-7}M	1.2×10^{-7}M
Co	5.03×10^{-8}M	5.0×10^{-8}M
Mo	1.00×10^{-7}M	1.0×10^{-7}M
Cu	1.96×10^{-8}M	2.0×10^{-8}M
Se	1.00×10^{-8}M	1.0×10^{-8}M
Cd	NA.	1.5×10^{-8}M

Figure S1 Dissolved Cu and Al concentration in surface water (original data are from Geotraces 2017 data product [*Mawji et al.*, 2015]). Although Cu concentration data are not available from the North Sea, it is assumed to be higher than that in the Southern Ocean, considering a larger input of dust in the North.

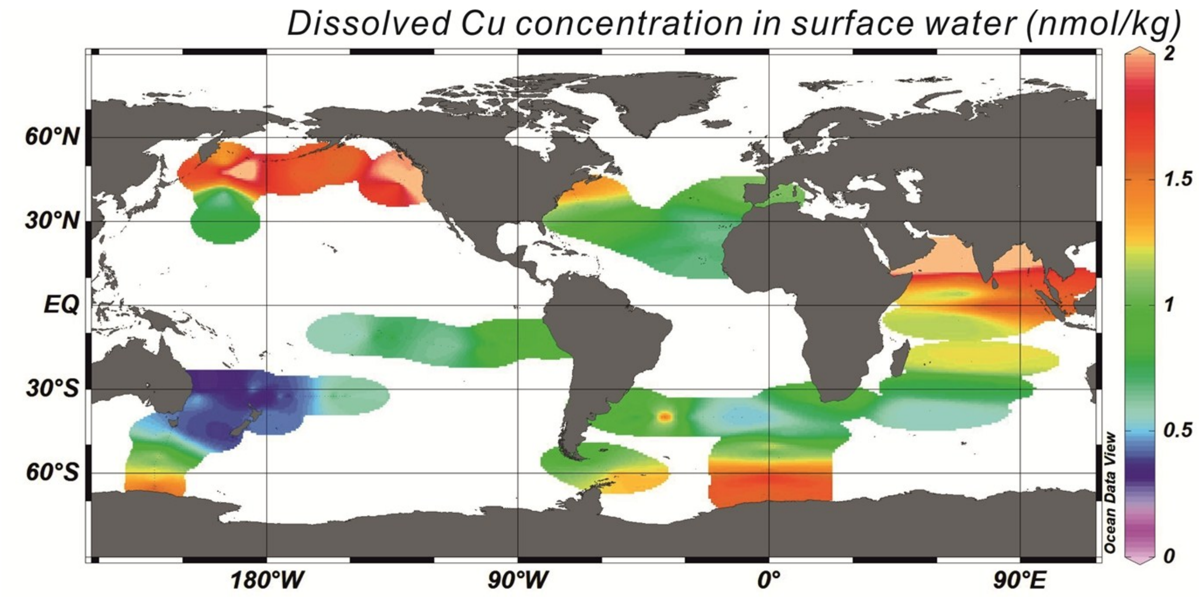

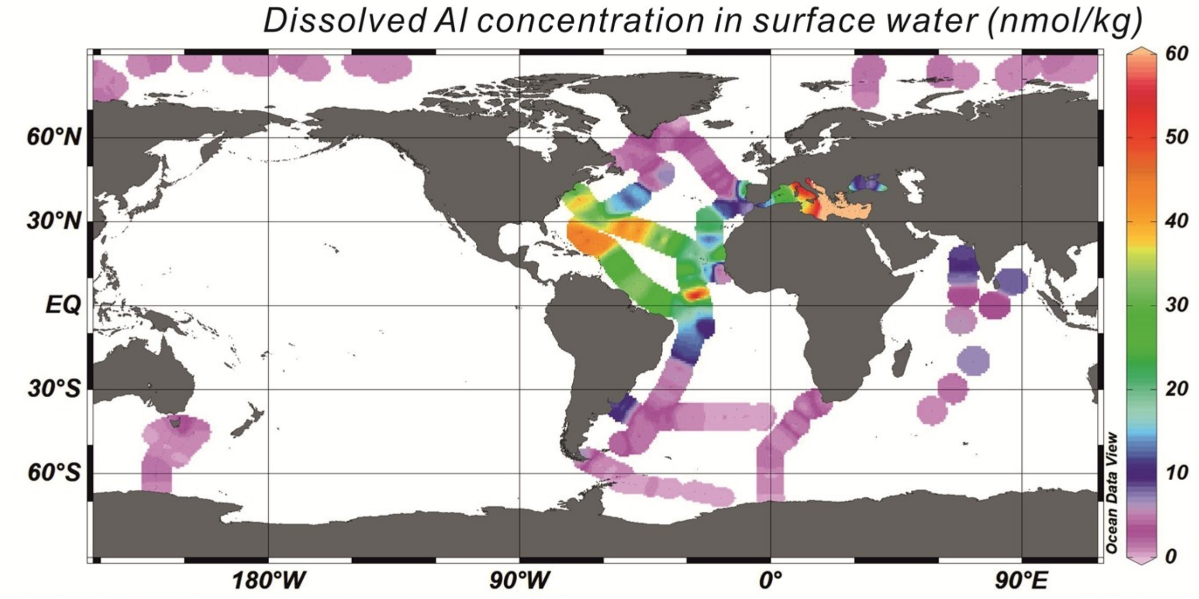

Mawji, E., et al. (2015), The GEOTRACES Intermediate Data Product 2014, *Marine Chemistry*, *177*, 1-8.
